# Disseminated Cryptococcosis in an Immunocompetent Child

**DOI:** 10.7759/cureus.15362

**Published:** 2021-05-31

**Authors:** Kalasekhar Vijayasekharan, Nitin Gupta, Suhas Reddisetti, Kavitha Saravu, Vishwapriya M Godkhindi

**Affiliations:** 1 Paediatric Haematology and Oncology, Manipal Comprehensive Cancer Care Centre, Manipal Academy of Higher Education, Manipal, IND; 2 Infectious Diseases, Kasturba Medical College, Manipal Academy of Higher Education, Manipal, IND; 3 Internal Medicine, Kasturba Medical College, Manipal Academy of Higher Education, Manipal, IND; 4 Oncopathology, Kasturba Medical College, Manipal Academy of Higher Education, Manipal, IND

**Keywords:** cryptococcus spp, cryptococcal lymphadenitis, pulmonary cryptococcosis, cryptococcosis. disseminated cryptococcosis, cryptococcosis

## Abstract

Disseminated cryptococcosis in children is a classic affliction associated with human immunodeficiency virus (HIV) infection or primary inherited immunodeficiency disorders (PID) with central nervous system being the most common site of dissemination. We report a rare case of disseminated cryptococcosis in an 11-year-old girl who presented with pulmonary involvement, hepatosplenomegaly, and generalized lymphadenopathy. No known inherited or acquired immune deficiencies were identified after a comprehensive laboratory work-up including genetic sequencing. She responded well to anti-fungal therapy (flucytosine and amphotericin followed by fluconazole) and is on regular follow-up.

## Introduction

Cryptococcosis is an invasive fungal infection reported from around the world [1], caused by *Cryptococcus neoformans *or *gattii *[2]. Although it primarily affects immunocompromised hosts, it is increasingly being reported from immunocompetent individuals as well. The common risk factors for *Cryptococcus *infection include human immunodficiency virus (HIV) infection, primary inherited immunodeficiency disorders (PID), immunosuppressive therapy, transplantation, malignancies, and idiopathic CD4 lymphopenia [2]. Pulmonary cryptococcosis in immunocompetent individuals is often self-limiting and hitherto rarely requires hospitalization [3]. In individuals with a history of immunosuppression, the fungus may disseminate to other organs, including the central nervous system. Reports of disseminated cryptococcosis in immunocompetent individuals are relatively rare. We report one such case of disseminated cryptococcosis in an immunocompetent child.

## Case presentation

An 11-year-old girl presented to the pediatric hematology-oncology out-patient department with complaints of intermittent high-grade fever and diffuse dull aching abdominal pain of one and half months duration. Ten days prior to the presentation, she developed progressive respiratory distress and yellowish discoloration of eyes. At presentation, she was hypoxic and required non-invasive ventilation to maintain saturation. On examination, she was found to have generalized lymphadenopathy involving bilateral cervical (Levels Ib, II, III, and IV) and axillary lymph nodes (anterior and central). She was also found to have hepatosplenomegaly. She had no signs of altered sensorium or meningeal irritation. Her birth and perinatal history was unremarkable with no past history of similar complaints, repeated infections or hospitalization. A provisional diagnosis of hemato-lymphoid malignancy was rendered awaiting further evaluation. 

**Figure 1:**
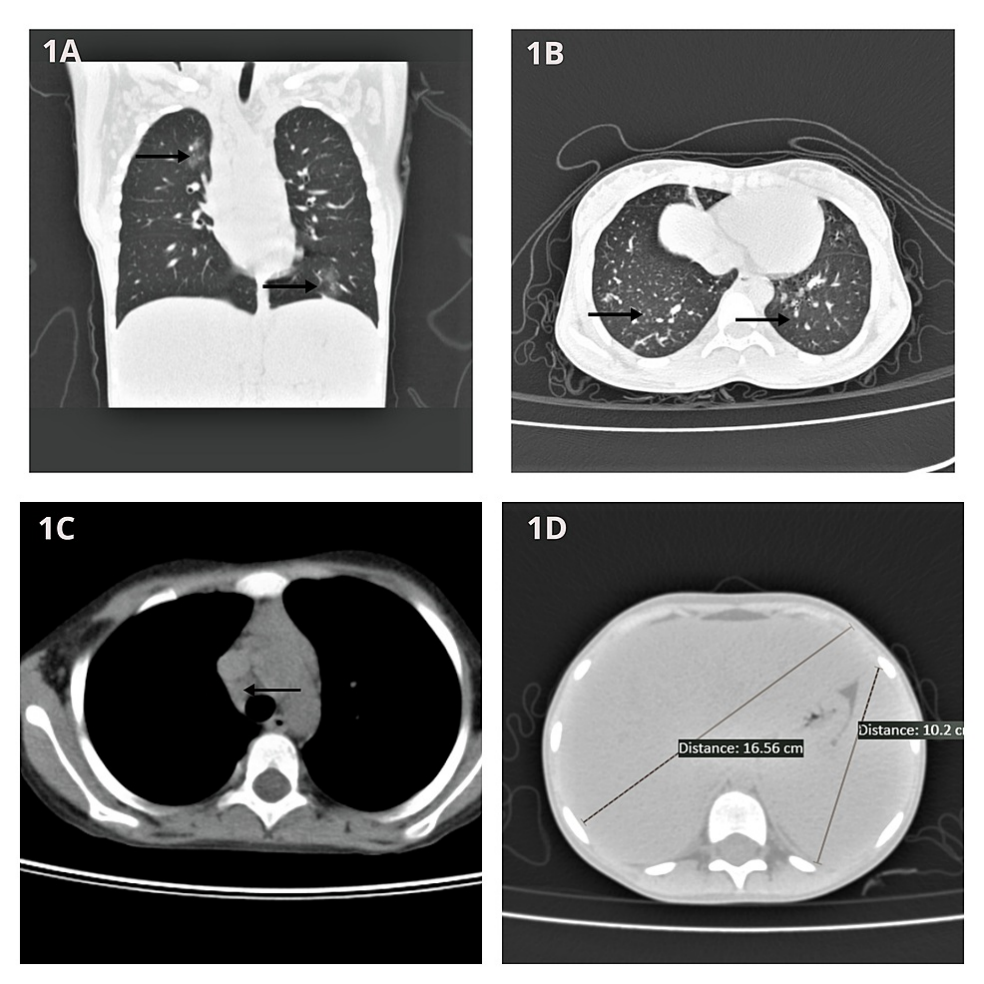
CECT of Thorax and Abdomen Bilateral areas of air-space opacifications with surrounding ground-glass opacities and interstitial thickening involving apical and anterior segments of the right upper lobe, along with antero-medial and basal segments of the left lower lobe (1A, 1B). Also noted were pretracheal and precarinal lymph nodes (1C). CECT abdomen shows hepatosplenomegaly (1D). CECT: Contrast-enhanced computed tomography

Her routine laboratory and biochemical parameters showed leukocytosis (total leucocyte count - 24,000/µL), increased serum bilirubin (total - 6.4 mg/dL, direct - 5.7 mg/dL), alkaline phosphatase (882 U/L), and elevated serum transaminases (aspartate transaminase - 115 IU/L, alanine transaminase - 49 IU/L). Contrast-enhanced computed tomography (CECT) of the chest revealed bilateral areas of air-space opacifications with surrounding ground-glass opacities and interstitial thickening involving apical and anterior segments of the right upper lobe, along with superior, antero-medial, and basal segments of the left lower lobe (Figures 1A, 1B). Also noted were pretracheal and precarinal lymph nodes (Figure 1C). CECT of the abdomen showed hepatosplenomegaly and multiple small retroperitoneal lymph nodes (Figure 1D).

**Figure 2:**
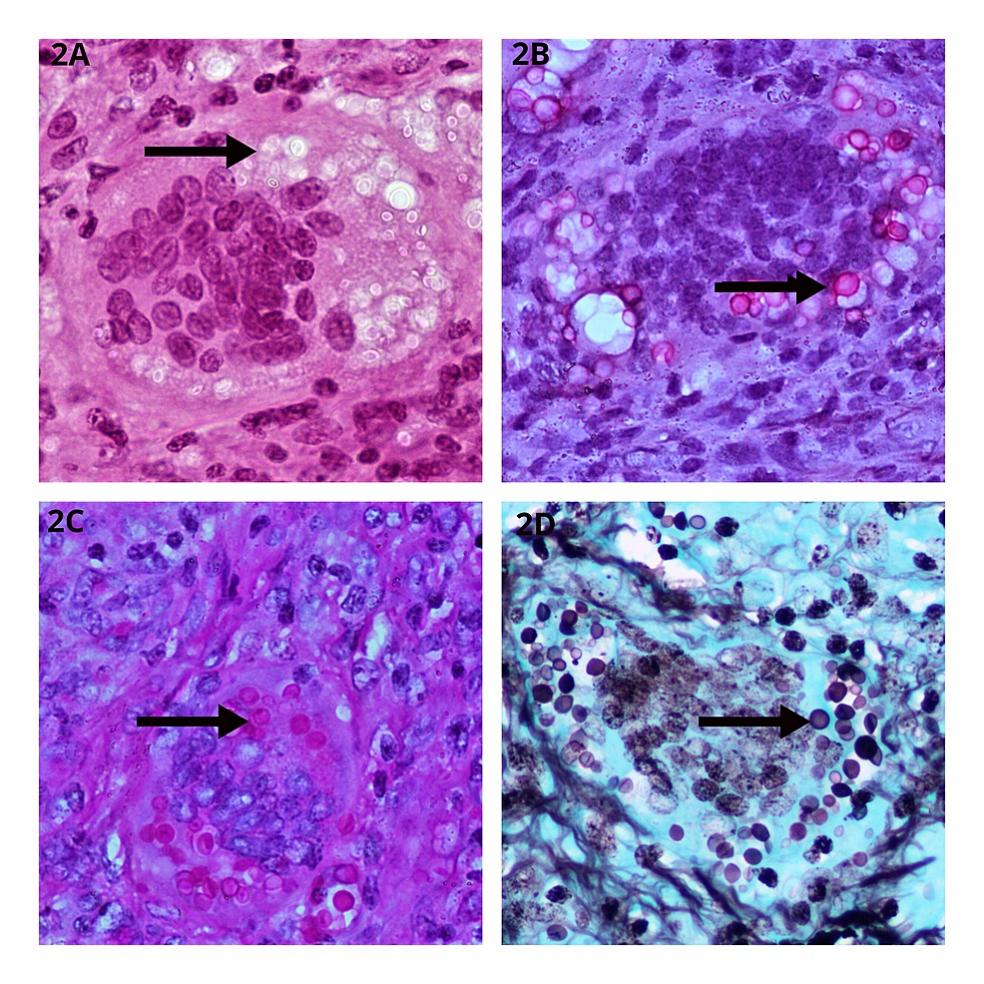
Histopathologic Examination of Left Axillary Lymph Node Biopsy. Haematoxylin and eosin stain (400x) showing multiple intracellular and extracellular capsulated yeast forms with foreign body type giant cell (2A). Mucicarmine (400x) stain highlights the mucopolysaccharide capsule (2B). Periodic Acid Schiff (400x) stain showing the magenta-pink stained thick-walled yeast forms (2C). Gomori’s methenamine silver (400x) stain showing the black stained yeast forms (2D).

The left axillary lymph node was biopsied and sent for histopathological evaluation, which showed non-caseating granulomas and presence of yeast-like forms of *Cryptococcus spp *(Figure 2). This was confirmed by mucicarmine staining which highlighted the mucopolysacchide capsule. A microbiological evaluation could not be rendered in view of limited nature of the biopsy specimen. 

A serum cryptococcal antigen lateral flow assay was done, which was also positive. Lumbar puncture was deferred due to the absence of headache and signs of meningeal irritation. She was found to be negative for HIV-1 and 2 by serology. Her immunoglobin levels (G, A, M) were normal. Her evaluation for chronic granulomatous disease by nitro blue tetrazolium (NBT) test was negative. Flow-cytometry lymphocyte subset analysis was unremarkable (CD45+ cells- 4900/µL, CD3+T cells-4081/µL, CD4+ cells-1919/µL, CD8+ cells-2044/µL, CD4/CD8 ratio-0.93, CD19+B cells-506/µL [10.3%], CD56+NK cells-294/µL [6%]). Targeted gene sequencing (Illumina® Inc., San Diego, California) for* CYBA*,* CYBB*, *NCF1*, *NCF2*, *NCF4*, *STAT3*, *DOCK8*, *IL12RB1*,* INFGR1*,* IL2RG*, *RAG1*, *RAG2*, *JAK3*, *IL7R*, *CD40*, *CD40LG*, *AICDA*, *UNG*, and *LIG4A* genes did not detect any pathogenic variants, thus effectively ruling out any inherited primary immunodeficiency syndromes. 

She was treated with liposomal amphotericin B (3 mg/kg) and flucytosine (100 mg/kg/day) for two weeks. Her fever and respiratory distress improved in five days. Her lymph nodes reduced significantly, and liver functions improved with the induction therapy. With improving clinical condition and laboratory parameters a repeat biopsy for serum cryptococcal antigen titre assay was deferred. She was discharged after two weeks of admission and continued on eight weeks of consolidation therapy with fluconazole (12 mg/kg). Her lymph nodes had completely regressed after the completion of consolidation therapy, and she was planned for one year of maintenance therapy with fluconazole (6 mg/kg). She is on regular follow-up and recuperating well. 

## Discussion

The mode of acquisition of cryptococcosis is by inhalation of the spores that get deposited in the smaller airways [4]. Most of the individuals with this early infection remain asymptomatic and it is self-limiting, but a small fraction of these patients may develop the symptomatic pulmonary disease [3,4]. Eventually, the fungus may disseminate from lungs to other sites of the body like the brain, peripheral lymph nodes, and skin. The determinants for the development of symptomatic or disseminated disease are the immune status of the host and the burden of inhaled spores. This is the reason why disseminated cryptococcosis is more common in patients with an impaired immune function such as HIV. In HIV-uninfected patients, other underlying condition that have been identified include liver cirrhosis, diabetes mellitus, and autoimmune diseases [5,6]. However, no immunosuppressive condition, diabetes mellitus, chronic kidney disease, steroid use, or close contact with pigeon or bird droppings was identified in our patient. 

The gold standard for diagnosis of cryptococcosis is either demonstration of the capsulated yeast forms or antigen detection test, either from the tissue/fluid or serum [7]. However, isolation of *Cryptococcus spp.* from a clinical specimen, or demonstration of typical capsular yeast forms on histological sections of tissues are equally helpful [7]. In our patient, cryptococcosis was not kept as a differential in the beginning, and therefore, only the histopathological specimens were sent, which demonstrated the typical budding capsulated yeast forms, and later confirmed on serum cryptococcal antigen lateral flow assay. 

Lumbar puncture was not done in our patient as she was immunocompetent without any neurological symptoms or signs suggestive of meningeal involvement [8]. Although mild to moderate cases of pulmonary cryptococcosis can be managed with oral azoles, induction therapy with amphotericin and flucytosine is required in cases with severe/disseminated disease [8]. Considering the severe pulmonary involvement and features suggestive of dissemination (hepatosplenomegaly, generalized lymphadenopathy), she was managed with intravenous amphotericin and flucytosine in the first two weeks, followed by eight weeks of fluconazole consolidation, and 12 months of maintenance therapy. The patient is on regular follow-up and is recuperating well.

## Conclusions

We report this rare case to highlight the possibility of disseminated cryptococcosis in immunocompetent patients presenting with pulmonary involvement, generalized lymphadenopathy, and hepatosplenomegaly without any known cause of immunosuppression or significant history of exposure in endemic areas. And lastly, highlight the need for a consolidated multidrug antifungal therapy, followed by a year-long maintenance therapy. 
